# Sulfonic Acid Derivative-Modified SBA-15, PHTS and MCM-41 Mesoporous Silicas as Carriers for a New Antiplatelet Drug: Ticagrelor Adsorption and Release Studies

**DOI:** 10.3390/ma13132913

**Published:** 2020-06-29

**Authors:** Michał Moritz, Małgorzata Geszke-Moritz

**Affiliations:** 1 Institute of Chemistry and Technical Electrochemistry, Faculty of Chemical Technology, Poznan University of Technology, Berdychowo 4, 60-965 Poznań, Poland; 2 Department of Pharmaceutical Chemistry, Faculty of Pharmacy, Poznan University of Medical Sciences, Grunwaldzka 6, 60-780 Poznań, Poland

**Keywords:** drug, silica, adsorption, release, carrier, modeling

## Abstract

Three mesoporous, siliceous materials, i.e., SBA-15 (Santa Barbara Amorphous), PHTS (Plugged Hexagonal Templated Silica) and MCM-41 (Mobil Composition of Matter), functionalized with a sulfonic acid derivative, were successfully prepared and applied as the carriers for the poorly water-soluble drug, ticagrelor. The siliceous carriers were characterized using nitrogen sorption analysis, X-ray diffraction (XRD), transmission electron microscopy (TEM) and elemental analysis. The adsorption studies were conducted in acetonitrile. At the highest equilibrium concentrations, the amount of ticagrelor *Q_e_* that adsorbed onto the examined silicas was in the range of 83 to 220 mg/g, increasing in the following order: PHTS-(CH_2_)_3_-SO_3_H < SBA-15-(CH_2_)_3_-SO_3_H < MCM-41-(CH_2_)_3_-SO_3_H. The equilibrium adsorption data were analyzed using the Freundlich, Jovanovich, Langmuir, Temkin, Dubinin-Radushkevich, Dubinin-Astakhov and Redlich-Peterson models. In order to find the best-fit isotherm for each model, a nonlinear fitting analysis was carried out. Based on the minimized values of the ARE function, the fit of the isotherms to the experimental points for ticagrelor adsorption onto the modified silicas can be ordered as follows: SBA-15-(CH_2_)_3_-SO_3_H (Redlich-Peterson > Dubinin-Astakhov > Temkin), PHTS-(CH_2_)_3_-SO_3_H (Redlich-Peterson > Temkin > Dubinin-Astakhov), MCM-41-(CH_2_)_3_-SO_3_H (Redlich-Peterson > Dubinin-Astakhov > Langmuir). The values of adsorption energy (above 8 kJ/mol) indicate the chemical nature of ticagrelor adsorption onto propyl-sulfonic acid-modified silicas. The results of release studies indicated that at pH 4.5, modified SBA-15 and MCM-41 carriers accelerate the drug dissolution process, compared to the dissolution rate of free crystalline ticagrelor. Intriguingly, modified PHTS silica provides prolonged drug release kinetics compared to other siliceous adsorbents and to the dissolution rate of crystalline ticagrelor. A Weibull release model was employed to describe the release profiles of ticagrelor from the prepared carriers. The time necessary to dissolve 50% and 90% of ticagrelor from mesoporous adsorbents at pH 4.5 increased in the following order: SBA-15-(CH_2_)_3_-SO_3_H < MCM-41-(CH_2_)_3_-SO_3_H < PHTS-(CH_2_)_3_-SO_3_H.

## 1. Introduction

**Figure 1:**
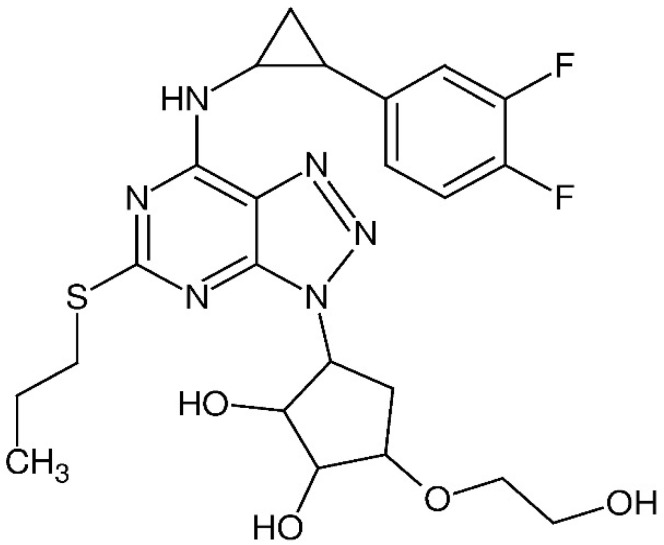
Chemical structure of ticagrelor.

Ticagrelor is the first cyclopentyltriazolopyrimidine antiplatelet agent approved for use in the European Union (European Medicines Agency in 2010) and the USA (Food and Drug Administration in 2011) [1]. The use of antiplatelet agents is of great importance in the therapy for patients with acute coronary syndromes. The chemical structure of ticagrelor [(1*S*,2*S*,3*R*,5*S*)-3-[7-{[(1*R*,2*S*)-2-(3,4-difluorophenyl)cyclopropyl]amino}-5-(propylsulfanyl)-3*H*-[1,2,3]triazolo[4,5-*d*]pyrimidin-3-yl]-5-(2-hydroxyethoxy)-1,2-cyclopentanediol] is presented in Figure 1.

It is an oral antagonist of the P2Y_12_ receptor. Ticagrelor is a rapid, selective, noncompetitive and reversible inhibitor of adenosine diphosphate-induced platelet aggregation [2]. Adenosine diphosphate is a key factor in thrombosis. It plays a significant role in platelet aggregation and adhesion via stimulation of the P2Y_12_ receptor. Some authors claim that ticagrelor has a dual mode of action [3]. It has been demonstrated that ticagrelor exerts an antiplatelet effect not only by antagonizing the P2Y_12_ receptor, but also by inhibiting adenosine uptake by the erythrocytes. The effect is mediated by the inhibition of the adenosine transporter, which provides protection for adenosine from intracellular metabolism [4,5].

Ticagrelor inhibits platelet aggregation in a dose-dependent manner [2]. The approved dosage regimen is a 180 mg loading dose (taken as two 90 mg tablets), then 90 mg twice daily [1,2]. Taken twice a day, ticagrelor leads to a biphasic increase in the plasma concentration over 24 h [6]. However, lower drug doses administered to patients with prior myocardial infarction were also examined [7]. Treatment with ticagrelor should be continued for up to 12 months [2].

In comparison with other antiplatelet agents such as clopidogrel and prasugrel, ticagrelor is a promising drug for the prevention of atherothrombotic events in adults suffering from acute coronary syndromes with high risk of ischemic events or who are unresponsive to clopidogrel [8]. Unlike clopidogrel and prasugrel, ticagrelor is not a prodrug [1]. It reveals satisfactory platelet inhibitory activity without the need of metabolic activation. However, it is extensively metabolized, and its major metabolite (AR-C124910XX) is also active and exhibits similar potency against the P2Y_12_ receptor [6]. The absorption of ticagrelor and its metabolite is rapid. Both substances exhibit linear pharmacokinetics [2]. The maximum plasma concentration is ca. 1.5 and ca. 2.5 h for ticagrelor and AR-C124910XX, respectively. However, after oral administration, the bioavailability of ticagrelor is only 36% [2]. The low oral bioavailability results from poor drug solubility and permeability. Ticagrelor is classified as Biopharmaceutics Classification System class IV [9]. Both ticagrelor and its metabolite are highly plasma-protein-bound (> 99%). Ticagrelor is metabolized mainly by CYP3A4 and CYP3A5, producing its major active metabolite AR-C124910XX [2]. The elimination of the drug and its metabolite occurs via hepatic metabolism and biliary secretion, respectively; they are excreted in feces (ca. 58%) and urine (ca. 27%). The elimination half-life is ca. 7 and ca. 8.5 h for ticagrelor and its metabolite, respectively [2]. 

Ticagrelor exhibits several adverse effects such as the risk of bleeding (including intracranial bleeding) [10], dyspnea and an increase in ventricular pauses of longer than 2.5 seconds [1,4,5,11]. Dyspnea is a very common ticagrelor side effect (> 10%). However, only in the case of persistent and intolerable ticagrelor-related dyspnea should drug discontinuation be considered [12]. Another unfavorable aspect of ticagrelor therapy is its cost. The price of ticagrelor-containing medicines (as it is a branded product) may be prohibitive for some patients, compared to generic clopidogrel [1]. Currently, experiments are focused on providing a more rapid ticagrelor antiplatelet effect. Parodi et al. reported the superiority of ticagrelor crushed pills versus integral tablets of equal dose in decreasing platelet reactivity in P2Y_12_-naive, ST-segment elevation-myocardial infarction patients undergoing primary percutaneous coronary intervention [13]. Earlier platelet inhibition of crushed pills compared with standard tablets probably resulted from enhanced drug absorption. The obtained results are of great importance for patients who are unable to swallow (sedated, intubated, with prior stroke or dysphagia). On the other hand, in order to decrease the dose of ticagrelor administered to patients, and thereby to diminish the side effects, it is essential to draw up drug delivery systems providing improved drug dissolution rates. In 2019, the group of Cho [9] fabricated ticagrelor-loaded nanostructured lipid carriers with improved drug oral bioavailability. The formulation prepared by the encapsulation of BCS class IV drug in lipid-based nanoparticles provided a sustained release profile and increased drug oral bioavailability, compared to raw ticagrelor [9]. In this area, mesoporous silicas seem to be promising carriers for poorly water-soluble drugs. Siliceous mesoporous molecular sieves were first introduced as drug delivery systems in 2001 [14]. Mesoporous silicas are characterized by large specific surface areas (usually several hundred m^2^/g), large pore volumes and with a pore diameter ranging from 2 to 50 nm [15]. Siliceous mesoporous molecular sieves are biocompatible [16] and can be easily modified using various functional groups [17]. Additionally, these materials exhibit chemical and hydrothermal stability [15]. All these features make mesoporous silicas attractive sorbents that can be used as the carriers in drug delivery systems providing controlled drug release. Since their first usage as carriers for ibuprofen [14], mesoporous silicas have been employed for other anti-inflammatory drugs [18,19], antihypertensive drugs [20,21], antibiotics [22,23], vitamins [24,25] and many others [26,27,28,29]. Controlled drug release may result from physical or chemical drug–silica interactions, or can be triggered by external stimuli such as magnetic fields [30], the presence of enzymes [31] or the presence of light [32]. In mesoporous silica-based drug delivery systems, the improvement of the dissolution rate of poorly water-soluble drugs is usually achieved by the transformation of the drug from a crystalline form to an amorphous state. This takes place during the adsorption of the drug molecules onto the large surface of the mesoporous carrier.

The aim of this study was to develop ticagrelor-mesoporous silica systems providing improved drug dissolution kinetics. Three types of mesoporous silicas exhibiting different textural properties, i.e., SBA-15, PHTS and MCM-41, were functionalized with propyl-sulfonic acid groups and used as carriers for a BCS class IV drug. SBA-15 silica possesses cylindrical mesopores [15]. Plugged, hexagonal templated silica (PHTS) reveals partially blocked mesoporous channels by amorphous particles (plugs) [15,33], whereas MCM-41 is characterized by small, uniform mesopores [34]. Depending on the synthesis conditions, the surface areas of SBA-15, PHTS and MCM-41 silicas vary from 500 to 950, 700 to 900 and 770 to 1500 m^2^/g, respectively [15]. The pore volumes for these materials reach values from 0.65 to 1.40, 0.65 to 0.80 and 0.60 to 1.20 cm^3^/g, respectively. Additionally, SBA-15 and PHTS silicas are characterized by the presence of micropores whose volumes range from 0.02 to 0.30 and 0.15 to 0.30 cm^3^/g, respectively. The pore walls of MCM-41 are thin, i.e., with a thickness ranging from 1.0 to 1.5 nm. Thus, this silica is characterized by low hydrothermal and chemical stabilities. In contrast, SBA-15 and PHTS silicas possess thick pore walls, i.e., of 3 to 6 nm, providing high hydrothermal stability [15]. It has been reported that among all of the analyzed silicas, PHTS possesses the highest mechanical stability [33]. The pore diameters of SBA-15, PHTS and MCM-41 vary from 5.0 to 8.0, 5.0 to 7.0 and 2.0 to 4.0 nm, respectively. All materials consist of hexagonally ordered mesopores with space group p6m for MCM-41 silica and p6mm for SBA-15 and PHTS materials [15,35]. 

It has been demonstrated that the functionalization of siliceous material with surface functional groups can increase the adsorption capacity of the mesoporous carrier. Additionally, the desired profile of drug release adsorbed on the siliceous surface can be achieved [36]. 

The three aforementioned mesoporous silicas were chosen as the adsorbents for ticagrelor to overcome the problem of its low dissolution rate. Amorphization is a very practical and low-cost technique which has attracted a great deal of interest. Using this technique, the crystalline drug is converted into its high energy amorphous form. The latter exhibits increased drug dissolution kinetics in comparison to the drug crystalline form [37]. Due to their large surface area, large pore volumes and pore diameters, mesoporous silicas are highly effective for drug amorphization. This is due to their ability to achieve the spatial confinement of drug molecules within their nanometer-scale pore structure [37]. Furthermore, due to their tunable pore size and surface area, the desired formulation can be obtained [37]. 

The process of ticagrelor adsorption was conducted in acetonitrile; meanwhile, drug release studies were performed in acetic buffer containing 0.5% (m/v) sodium dodecyl sulfate. To better understand the mechanisms of ticagrelor-siliceous surface interactions, both the adsorption modeling and release process modeling are provided. The sets of adsorption isotherm parameters, as well as the release experimental points, were assessed using nonlinear fitting analysis and the average relative error (ARE) function. The equilibrium adsorption data were analyzed using the Freundlich, Jovanovich, Langmuir, Temkin, Dubinin-Radushkevich, Dubinin-Astakhov and Redlich-Peterson adsorption isotherm models, whereas the release experimental data were examined using the Weibull model. We believe that the results of the present investigation will allow us to find an effective mesoporous carrier for ticagrelor.

## 2. Materials and Methods 

### 2.1. Reagents and Materials

Ticagrelor (99.1%) was supplied from Polpharma (Starogard Gdański, Poland). Mesoporous silica MCM-41, tetraethoxysilane (TEOS) (≥ 99.0%), (3-mercaptopropyl)trimethoxysilane (MPTMS) (95%), triblock copolymer poly(ethylene glycol)-block-poly(propylene glycol)-block-poly(ethylene glycol) (Pluronic® P123, average molar weight 5800), anhydrous toluene (99.8%), hydrochloric acid (≥ 32.0%), sodium acetate (99%), acetic acid (99.7%) and sodium dodecyl sulfate (SDS) were obtained from Sigma-Aldrich (Poznań, Poland). Acetonitrile (> 99.5%), 2-propanol (≥ 99.7%), chloroform (≥ 99.8%) and hydrogen peroxide (30%) were purchased from Avantor Performance Materials Poland (Gliwice, Poland).

### 2.2. Preparation of Mesoporous Adsorbents

#### 2.2.1. SBA-15 Synthesis

SBA-15 was synthesized according to the method described in [38] with modifications. Initially, 48.0 g of amphiphilic triblock copolymer was mixed with 1800 cm^3^ of aqueous hydrochloric acid (1.6 mol/dm^3^) and maintained at 35 °C. Next, 102.0 g of TEOS was added and the solution was stirred at 35 °C for 24 h. Subsequently, the reaction mixture was refluxed at 100 °C for 24 h. After filtration and washing with distilled water, the white solid was dried in the air. The resulting solid phase was calcined at 500 °C for 6 h.

#### 2.2.2. PHTS Synthesis

PHTS material was prepared according to the method described in [33] with modifications. In brief, 24.0 g of triblock copolymer P123 was dispersed in 900 cm^3^ of aqueous HCl (1.6 mol/dm^3^). Next, to the reactant mixture was heated at 60 °C and 90.0 g of tetraethoxysilane was added. The solution was stirred for 8 h at 60 °C, followed by aging at 80 °C for 16 h. After filtration and washing with distilled water, the solid phase was dried in air and calcined at 500 °C for 6 h.

### 2.3. Functionalization of Siliceous Matrices

Modified SBA-15, PHTS and MCM-41 samples were prepared using the grafting strategy. In brief, 4000 g of crude sample (first dried at 120 °C for 6 h) was dispersed in 50 cm^3^ of dry toluene containing (3-mercaptopropyl)trimethoxysilane (0.27 mol/dm^3^). The mixture was refluxed at 100 °C for 24 h. Then, the MPTMS-modified silicas were filtered. The final product was washed several times with dry toluene and chloroform. The obtained solid was dried at 45 °C for 1 h and then at 70 °C for 20 h. The final stage of the procedure consisted of the oxidation of the mercaptopropyl groups present at the silica surface to propyl-sulfonic acid functions. For this purpose, the MTPMS-functionalized material was mixed with 80 cm^3^ of H_2_O_2_ (~30% w/v). The oxidation process took place at 25 °C for 24 h. Then, the resultant material was washed using a Büchner funnel. First, the solid was dried at 35 °C for 20 h and then at 70 °C for 24 h. Functionalized mesoporous silicas were designated as SBA-15-(CH_2_)_3_-SO_3_H, PHTS-(CH_2_)_3_-SO_3_H and MCM-41-(CH_2_)-SO_3_H.

### 2.4. Ticagrelor Adsorption Experiments

The initial ticagrelor adsorption experiments onto parent and propyl-sulfonic acid-modified matrices were performed in acetonitrile and 2-propanol. The initial drug concentration was 2000 mg/dm^3^. In a typical experiment, 0.050 g of silica was suspended in 5 cm^3^ of acetonitrile or 2-propanol with an appropriate concentration of ticagrelor. The studies were conducted under stirring at 23 °C for 24 h. The amount of ticagrelor adsorbed at the siliceous surface Qe (mg/g) and the efficiency of adsorption *E_ads_* (%) were calculated using Equations (1) and (2), respectively:

(1) $$Q_{e}=\frac{(C_{0}-C_{e})\times V}{m}$$

(2) $$E_{ads}=\left(\frac{C_{0}-C_{e}}{C_{0}}\right)\times 100\%$$

where *C*_0_ (mg/dm^3^) and *C*_e_ (mg/dm^3^) are the initial and equilibrium ticagrelor concentration, respectively, *V* (dm^3^) is the volume of ticagrelor solution and *m* (g) represents the mass of siliceous adsorbent.

The equilibrium concentration of ticagrelor was determined spectrophotometrically at the analytical wavelength of 254 nm. Prior the measurement, the suspension was centrifuged at 3500× *g* for 15 min, and the supernatant was diluted with an appropriate volume of solvent.

In comparison to parent silicas, the preliminary studies revealed better adsorption efficiency of ticagrelor using the modified sorbents. Moreover, the adsorption process was more efficacious for experiments performed from acetonitrile as compared to 2-propanol. Thus, a detailed analysis of drug adsorption was conducted from the former employing propyl-sulfonic acid-functionalized silicas as the adsorbent. The adsorption conditions were as described above, whereas ticagrelor initial concentrations were in the range of 250 to 7030 mg/dm^3^.

### 2.5. Modeling of Asorption Eperimental Data

The equilibrium data obtained in adsorption studies were analyzed using Freundlich, Jovanovich, Langmuir, Temkin, Dubnin-Radushkevich, Dubinin-Astakhov and Redlich-Peterson equations [39,40]:

(3) $$Q_{e}=K_{F}\times C^{1/n_{F}}$$

(4) $$Q_{e}=Q_{J(\max)}\times \left[1-\exp\left(-K_{J}\times C_{e}\right)\right]$$

(5) $$Q_{e}=\frac{Q_{L(\max)}\times K_{L}\times C_{e}}{1+K_{L}\times C_{e}}$$

(6) $$Q_{e}=\frac{RT}{b_{T}}\ln(K_{T}\times C_{e})$$

(7) $$Q_{e}=Q_{DR(\max)}\exp\left\{-K_{DR}{\left[RT\ln\left(\frac{C_{s}}{C_{e}}\right)\right]}^{2}\right\}$$

(8) $$Q_{e}=Q_{DA(\max)}\exp\left\{-K_{DA}{\left[RT\ln\left(\frac{C_{s}}{C_{e}}\right)\right]}^{n_{DA}}\right\}$$

(9) $$Q_{e}=\frac{K_{RP}\times C_{e}}{1+a_{RP}\times C_{e}{}^{\beta }}$$

where *Q_e_* (mg/g) and *C_e_* (mg/g) represent the equilibrium amount of adsorbed ticagrelor and its equilibrium concentration, respectively; *K_F_* (mg^1-1/n^ dm^3/n^/g) is the Freundlich constant, *n_F_* describes the exponential constant of Freundlich model; *K_J(max)_* (mg/g) represents the maximum adsorption capacity estimated from Jovanovich equation, *K_J_* (dm^3^/mg) is the Jovanovich constant; *Q*_*L*(*max*)_ (mg/g) is the maximum adsorption capacity determined from the Langmuir equation, *K_L_* (dm^3^/mg) represents the Langmuir constant; *K_T_* (dm^3^/mg) represents the Temkin equilibrium binding constant, *b_T_* (J g/mol mg) is the Temkin constant related to the adsorption heat, *R* (8.314 J/mol K) is the gas constant, T (K) is the absolute temperature; *Q*_*DR*(*max*)_ (mg/g) is the maximum adsorption capacity estimated from Dubinin-Radushkevich model, *K_DR_* (mol^2^/J^2^) represents a constant related to the sorption energy, *C_s_* (mg/dm^3^) is the solubility of the adsorbate; *Q*_*DA*(*max*)_ (mg/dm^3^) represents the maximum adsorption capacity estimated from Dubinin-Astakhov model, *K_DA_* (mol*^nDA^*/J*^nDA^*) is the isotherm constant related to the sorption energy, *n_DA_* is the heterogeneity factor of Dubinin-Astakhov isotherm; *K_RP_* (dm^3^/g) and *a_RP_* (dm^3*β*^/mg*^β^*) are the Redlich-Peterson constants, and *β* represents the exponential constant of Redlich Peterson isotherm.

The adsorption isotherms based on the Polanyi potential (Dubinin-Radushkevich and Dubinin-Astakhov equations) enable the calculation of mean free energy of adsorption [41,42,43]:

(10) $$E_{DR}=\frac{1}{\sqrt{2\times K_{DR}}}$$

(11) $$E_{DA}=\frac{1}{\sqrt{2}\times \sqrt[{n_{DA}}]{K_{DA}}}$$

where *E_DR_* and *E_DA_* represent the adsorption energy (J/mol) calculated from Dubinin-Radushkevich and Dubinin-Astakhov models, respectively; *K_DR_* (mol^2^/J^2^) and *K_DA_* (mol^nDA^/J^nDA^) describe the constant related to the energy of adsorption for given isotherm (Equations (7) and (8)) and *n_DA_* is a heterogeneity factor occurring in Dubinin-Astakhov isotherm.

In order to find the most suitable parameters for the isotherm equations, the average relative error (ARE) function was used. The ARE error function can be written as follows [43]:

(12) $$\mathrm{ARE}=\frac{100}{n}{\sum_{i=1}^{n}\left|\frac{Q_{e,\exp.}-Q_{e,calc.}}{Q_{e,\exp.}}\right|}_{i}$$

where *Q_,calc._* and *Q_e,exp._* represent the calculated and experimental amount of adsorbed active substance, mg/g, respectively and *n* denotes the number of measurement points.

The parameters in Equations (3)–(9) were assessed using nonlinear fitting analysis approaching to the minimization of the value of average relative error (ARE). The optimization procedure was performed employing the solver add-in with Microscoft® Excel 2007 Software (Redmond, WA, USA).

### 2.6. Preparation of Dosage Forms

Siliceous samples containing ticagrelor were prepared as follows: 2000 g of modified mesoporous silica was suspended in 0.2 dm^3^ of ticagrelor solution (2000 mg/dm^3^) in acetonitrile. The process occurred under stirring at 23 °C for 24 h. After filtration, the obtained carrier loaded with ticagrelor was dried at 50 °C for 24 h. The formulations loaded with ticagrelor were designated as SBA-15-(CH_2_)_3_-SO_3_H+TGR, PHTS-(CH_2_)_3_-SO_3_H+TGR and MCM-41-(CH_2_)_3_-SO_3_H+TGR. The amount of adsorbed ticagrelor was determined from the elemental analysis, taking into account the quantity of nitrogen present in the analyzed carrier.

### 2.7. Ticagrelor Release Studies

Ticagrelor release studies from mesoporous carriers were carried out in acetic buffer with 0.5% (m/v) sodium dodecyl sulfate (SDS). This buffer is the recommended dissolution medium for the determination of the dissolution kinetics of active substances characterized by low water solubility [44,45,46,47]. The usage of this medium creates conditions analogous to those occurring in the stomach after eating. The composition of the buffer fulfilled the requirements of [46] with modifications. Initially, 0.2 g of drug-loaded siliceous carrier was dispersed in 1 dm^3^ of dissolution medium. The experiments were carried out at 23 °C under magnetic stirring. In brief, at suitable time intervals (from 15 min to 24 h), an aliquot of 1.5 cm^3^ was taken and centrifuged (13,000 rpm, 5 min). Next, the supernatant was diluted with dissolution medium. The amount of ticagrelor released from the carrier was assessed using the spectrophotometric method (analytical wavelength of 254 nm).

### 2.8. Ticagrelor Release Modeling

The ticagrelor release profiles were analyzed using the Weibull model, which can be expressed as [48,49]:

(13) $$F=1-\exp\left(-\frac{t^{b}}{a}\right)$$

where *a* and *b* describe the scale and the shape parameter of Weibull release model, respectively; *F* represents the fraction (takes the value from 0 to 1) of drug released at time *t* (h).

The parameters of the Weibull model were estimated using nonlinear fitting analysis. As the criterion of the Weibull equation fit with the experimental data, the ARE function was employed. However, in comparison to the determination of the parameter values of adsorption isotherms, in this case, the function can be expressed as follows:

(14) $$\mathrm{ARE}=\frac{100}{n}{\sum_{i=1}^{n}\left|\frac{F_{\exp.}-F_{calc.}}{F_{\exp.}}\right|}_{i}$$

where *F_calc._* and *F_exp._* describe the calculated and experimental fraction of released active substance and *n* denotes the number of measurement points.

The Weibull model also makes it possible to assess the time needed for the release of a certain amount of active substance from prepared dosage forms. In the case of a release of 50% (*F* = 0.5) and 90% (*F* = 0.9) of active substance, the time can be calculated using the following equations, respectively [50]:

(15) $$t_{50\%}=10^{\frac{\log a-0.159}{b}}$$

(16) $$t_{90\%}=10^{\frac{\log a+0.362}{b}}$$

### 2.9. Instrumentation

Adsorption–desorption isotherms for nitrogen were measured at −196 °C with an Autosorb iQ analyzer (Quantachrome Instruments, Boynton Beach, FL, USA). Before analysis, the materials were degassed at 100 °C for 12 h. The specific surface area was determined from the Brunauer-Emmet-Teller (BET) method in a relative pressure (p/p0) range of 0.09 to 0.30. The average pore diameter and volume were calculated from the isotherm desorption branch using Barret-Joyner-Hallenda (BJH) mode.

Transmission electron microscopy (TEM) was done on a JOEL JEM 1200 EX electron microscope (JOEL, Tokyo, Japan) operated at an acceleration voltage of 80 kV.

Spectrophotometric analyses were performed on a Beckman DU 7500 spectrophotometer (Beckman, Fullerton, CA, USA).

Elemental analysis (C, H, N, S) was carried out using Elementar Vario EL III Elemental Analyser (Elementar Vario, Hanau, Germany).

The release studies were conducted using the Electrolab Dissolution Tester EDT-08Lx (Electrolab, Mumbai, India).

The differential scanning calorimetry (DSC) analyses were done in a nitrogen atmosphere on a DSC-8500 Perkin-Elmer Instrument (Perkin-Elmer, Waltham, MA, USA) at the temperature range from 30 to 200 °C (heating rate of 10 °C /min).

## 3. Results and Discussion

### 3.1. Characterization of Mesoporous Adsorbents

The results of the surface analysis for pure and sulfonic acid derivative-modified mesoporous silicas are presented in Table 1 and Table 2, respectively. 

**Figure 2:**
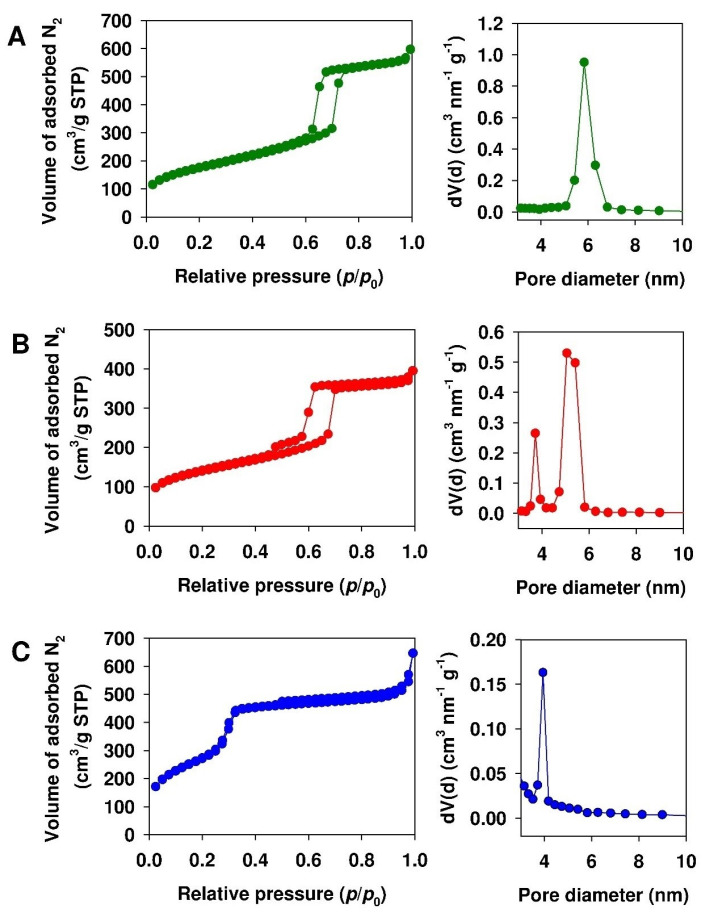
Nitrogen adsorption–desorption isotherms of (**A**) SBA-15-(CH_2_)_3_-SO_3_H, (**B**) PHTS-(CH_2_)_3_-SO_3_H, (**C**) MCM-41-(CH_2_)_3_-SO_3_H mesoporous adsorbents. On the right, the pore size distribution of silicas is presented.

The modification process of SBA-15, PHTS and MCM-41 materials resulted in the reduction of surface area and pore volume by 21.7 and 12.4%, 28.5% and 21.4%, and 9.0 and 43.9%, respectively. Moreover, the modification process resulted in a significant reduction of the microporous fraction of SBA-15 and PHTS silicas, i.e., reaching 47.5% and 36.5%, respectively. The nitrogen adsorption–desorption isotherms of propyl-sulfonic acid-functionalized mesoporous silicas with corresponding pore size distribution are presented in Figure 2.

According to IUPAC nomenclature, all of the nitrogen adsorption–desorption isotherms followed the type IV isotherm [51]. The observed hysteresis loop is characteristic of capillary condensation within uniform pores. The prepared mesoporous materials revealed different shapes and positions of the hysteresis loop, associated with the specific mesopore geometries. The obtained values of the textural parameters are typical for this kind of material [15]. As shown in Figure 2A, the nitrogen sorption isotherm of SBA-15-(CH_2_)_3_-SO_3_H revealed a H1-type hysteresis loop at the relative pressure range of 0.60 to 0.76, which is characteristic of cylindrical-like, pore structures. The isotherm exhibited sharp adsorption and desorption branches, confirming the narrow pore size distribution [52]. The nitrogen sorption isotherm of PHTS-(CH_2_)_3_-SO_3_H silica revealed the hysteresis loop at the relative pressure range of 0.45 to 0.71. It is worth mentioning that PHTS exhibited a characteristic two-step desorption branch (bimodal pore size distribution). The two-step evaporation of the condensate arose from the partially blocked mesoporous channels by amorphous silica nanoparticles [33]. The nitrogen adsorption–desorption isotherm of MCM-41-(CH_2_)_3_-SO_3_H mesoporous silica revealed the hysteresis loop at a relative pressure range of 0.25 to 0.33. It emerged that at higher pressures (exceeding 0.25 *p/p*_0_), the adsorption in mesopores results in a multilayer arrangement until condensation occurs. This can be illustrated by a sharp increase in the adsorption volume. After the mesopores are filled up, adsorption occurs at the external surface [53]. It should be mentioned that in the structures of SBA-15-(CH_2_)_3_-SO_3_H and PHTS-(CH_2_)_3_-SO_3_H silicas, the presence of a microporous area characterized by a volume of 0.054 and 0.073 cm^3^/g, respectively, can be distinguished. The pore volume and diameter of the mesoporous adsorbents decreased in the following order: SBA-15-(CH_2_)_3_-SO_3_H > PHTS-(CH_2_)_3_-SO_3_H > MCM-41-(CH_2_)_3_-SO_3_H. As mentioned, the modified MCM-41 silica was characterized by the largest surface area and the smallest pore volume and diameter among all analyzed materials.

The number of functional groups for the modified silicas was estimated based on the results of the elemental analysis (i.e., sulfur content). The functional group content was in the range of 2.03 × 10^−4^ to 5.72 × 10^−4^ mol/g. It should be mentioned that PHTS-(CH_2_)_3_-SO_3_H silica revealed the lowest number of functional groups. 

**Figure 3:**
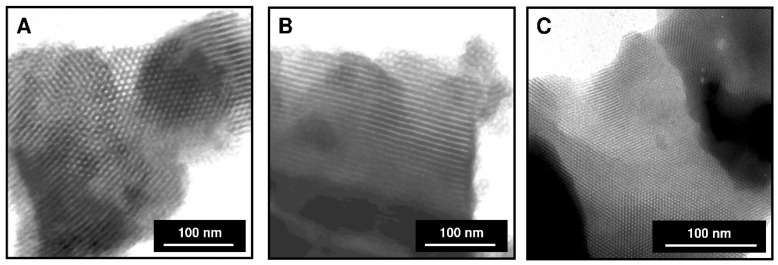
TEM micrographs of (**A**) SBA-15-(CH_2_)_3_-SO_3_H, (**B**) PHTS-(CH_2_)_3_-SO_3_H, (**C**) MCM-41-(CH_2_)_3_-SO_3_H mesoporous adsorbents.

Figure 3 shows the TEM micrographs of synthesized mesoporous silicas. Figure 3A displays a hexagonal channel array (honeycomb-like structure) of SBA-15-(CH_2_)_3_-SO_3_H silica [15]. Figure 3B exhibits the parallel mesoporous channel arrangement of PHTS-(CH_2_)_3_-SO_3_H [9]. Meanwhile, Figure 3C illustrates the hexagonal pore arrangement of MCM-41-(CH_2_)_3_-SO_3_H sample [15].

### 3.2. Adsorption Studies

**Figure 4:**
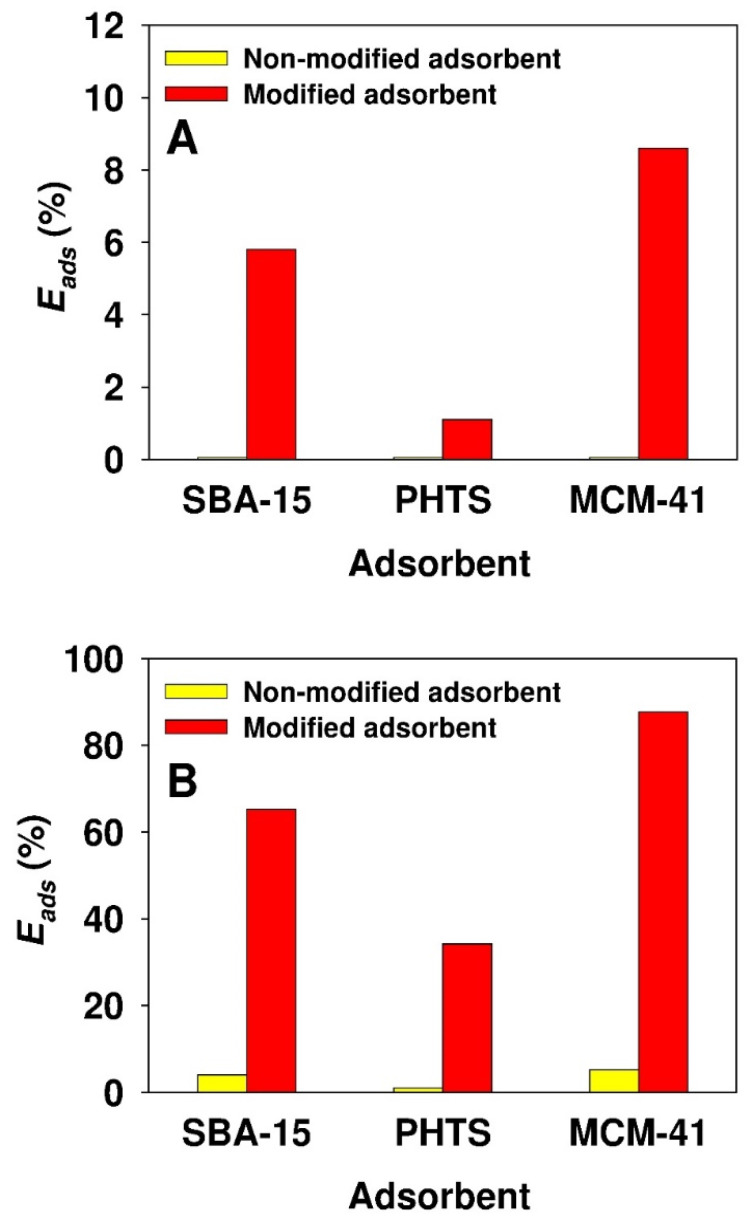
Comparison of ticagrelor adsorption efficiency in (**A**) 2-propanol and (**B**) acetonitrile onto nonmodified and propyl-sulfonic acid-modified mesoporous silicas. Initial ticagrelor concentration *C*_0_ = 2000 mg/dm^3^.

Figure 4 shows the results of preliminary adsorption studies of ticagrelor at a concentration of 2000 mg/dm^3^ onto nonmodified and propyl-sulfonic acid-modified mesoporous silicas in two solvents, i.e., acetonitrile and 2-propanol. For experiments conducted in 2-propanol, the drug adsorption efficiency on unmodified adsorbents was minimal, whereas on modified sorbents, it was in the range of 1.1 to 8.6%. For experiments conducted in acetonitrile, the adsorption efficiency was in the range of 1.0 to 5.2% and 34.2 to 87.7% for the parent and modified silicas, respectively. Interestingly, for both parent and modified silicas, the lowest and the highest values of adsorption efficiency were noted for PHTS and MCM-41 silicas, respectively.

The obtained data regarding adsorption efficiency clearly indicate that the process of ticagrelor adsorption is favored in acetonitrile using propyl-sulfonic acid-modified silicas. For this reason, further adsorption studies were conducted in this aprotic solvent using functionalized mesoporous molecular sieves as the adsorbents. A similar trend of favored adsorption in aprotic solvent was noted in our previous studies concerning the adsorption of chlorhexidine [54], boldine [55] and niacinamide [24] onto siliceous matrices.

**Figure 5:**
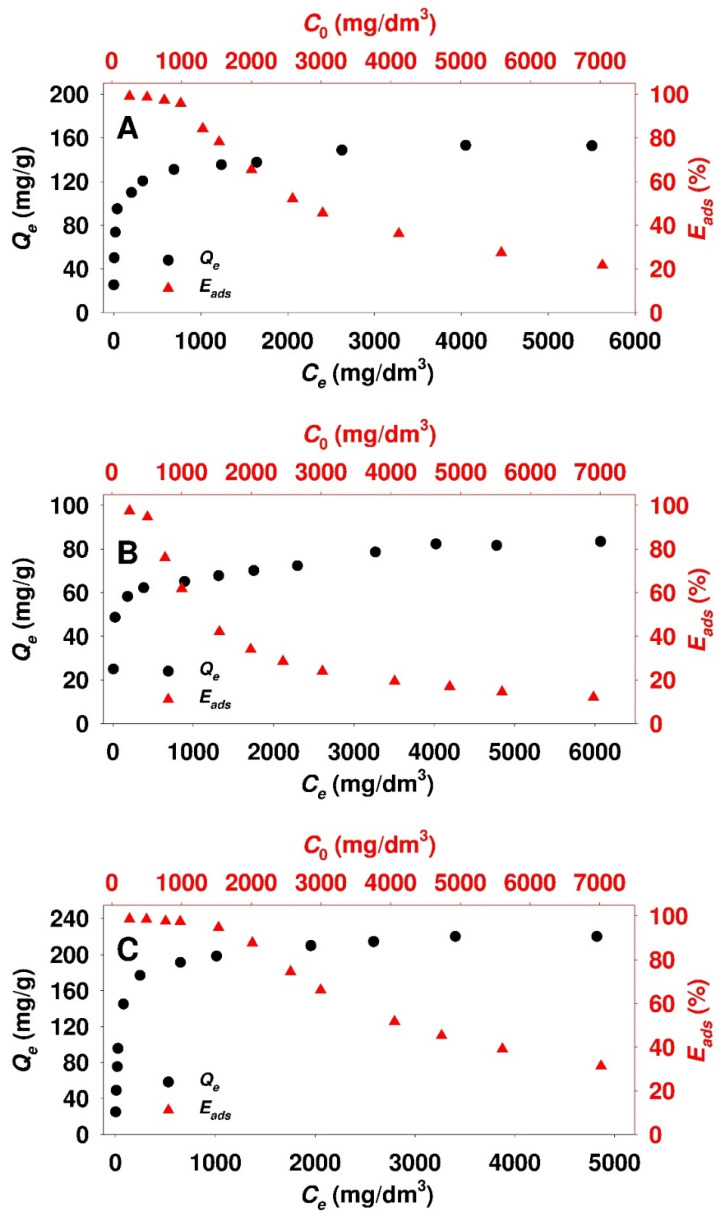
Adsorption isotherms and efficiency of ticagrelor in acetonitrile medium onto: (**A**) SBA-15-(CH_2_)_3_-SO_3_H, (**B**) PHTS-(CH_2_)_3_-SO_3_H and (**C**) MCM-41-(CH_2_)_3_-SO_3_H mesoporous adsorbents.

Figure 5 shows the adsorption isotherms and the efficiencies of ticagrelor onto modified silicas.

The shape of all isotherms with a distinct, steeply rising branch evolving into a plateau indicates the type of adsorption in accordance with Langmuir model [56]. At the highest equilibrium concentrations (i.e., at the plateau), the amount of adsorbed ticagrelor *Q_e_* onto examined silicas was in the range of 83 to 220 mg/g, and increased in the following order: PHTS-(CH_2_)_3_-SO_3_H < SBA-15-(CH_2_)_3_-SO_3_H < MCM-41-(CH_2_)_3_-SO_3_H. At the lowest initial drug concentrations, the adsorption efficiency for all materials was very high, exceeding 97%. With the increase of the initial adsorbate concentration, the adsorption efficiency decreased, reaching the lowest values, i.e., 12, 22 and 32%, for PHTS-(CH_2_)_3_-SO_3_H, SBA-15-(CH_2_)_3_-SO_3_H and MCM-41-(CH_2_)_3_-SO_3_H silicas, respectively.

### 3.3. Estimation of Isotherm Parameters Using Nonlinear Fitting Analysis

The experimental points presented in Figure 5 have been used to determine the equations precisely describing the adsorption isotherms. The parameters of the adsorption isotherms determined from the nonlinear fitting analysis based on Equations (3)–(9) for propyl-sulfonic acid-functionalized mesoporous samples are presented in Table 3.

**Figure 6:**
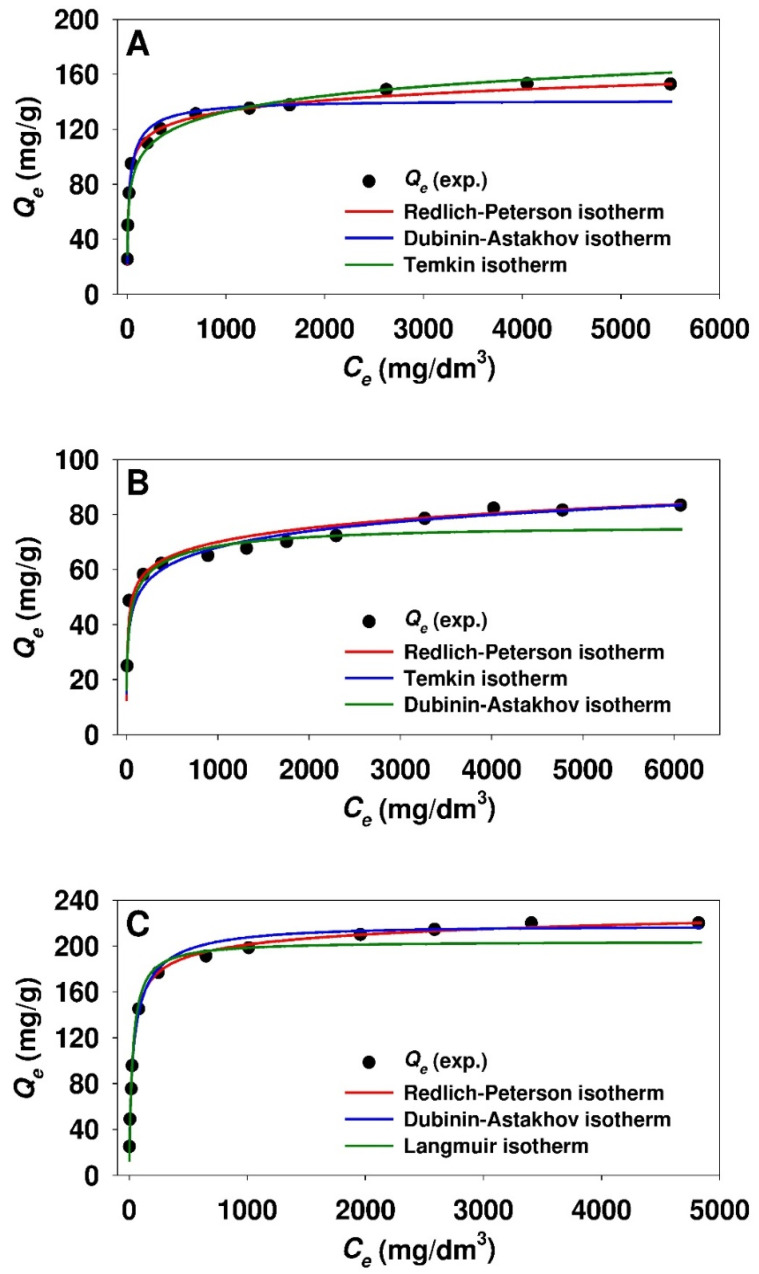
Comparison of experimental and predicted isotherms of ticagrelor adsorption onto: (**A**) SBA-15-(CH_2_)_3_-SO_3_H, (**B**) PHTS-(CH_2_)_3_-SO_3_H and (**C**) MCM-41-(CH_2_)_3_-SO_3_H mesoporous adsorbent. The best fitted isotherm models derived from nonlinear analysis are presented.

Based on the minimized values of ARE function, the fit of the isotherms to the experimental points for ticagrelor adsorption onto modified silicas (see Table 3) can be arranged as follows: SBA-15-(CH_2_)_3_-SO_3_H (Redlich-Peterson > Dubinin-Astakhov > Temkin), PHTS-(CH_2_)_3_-SO_3_H (Redlich-Peterson > Temkin > Dubinin-Astakhov), MCM-41-(CH_2_)_3_-SO_3_H (Redlich-Peterson > Dubinin-Astakhov > Langmuir). A comparison of the experimental equilibrium data and predicted adsorption isotherms (for best-fitted models) for ticagrelor onto the examined silicas is presented in Figure 6.

Depending on the employed adsorption model, the maximum adsorption capacity of the modified silicas determined based on the Langmuir, Dubinin-Radushkevich and Dubinin-Astakhov equations was in the range of 70.6 to 78.2, 136.6 to 156.5 and 204.1 to 240.7 mg/g for PHTS-(CH_2_)_3_-SO_3_H, SBA-15-(CH_2_)_3_-SO_3_H and MCM-41-(CH_2_)_3_-SO_3_H silica, respectively. It should be noted that the highest maximum adsorption capacity values (i.e., the upper limit of the presented ranges) were ascribed to the Dubinin-Radushkevich isotherm. Despite being the same modification process, propyl-sulfonic acid-modified MCM-41 silica revealed a 3-fold and 1.5-fold higher adsorption capacity value compared to PHTS and SBA-15, respectively. The value of adsorption capacity corresponds to the content of sulfonic groups (degree of modification). As demonstrated in Table 2, the PHTS sample showed the lowest content of surface functional groups, although it was characterized by a surface area similar to SBA-15 silica. This may result from the presence of silica particles inside the mesoporous channels of PHTS material (steric hindrance).

The Jovanovich, Langmuir, Dubinin-Radushkevich and Dubinin-Astakhov models allowed us calculate the maximum adsorption capacities *Q*_*ads*(*max*)_ for the examined sorbents. Based on the *Q*_*ads*(*max*)_ value, the surface area-normalized maximum adsorption capacity *Q*_*s*(*max*)_ (mg/m^2^) was calculated using the following Equation [54]:

(17) $$Q_{s(\max)}=\frac{Q_{ads(\max)}}{S_{BET}}$$

where *S_BET_* (m^2^/g) represents the specific surface area of siliceous adsorbent.

**Figure 7:**
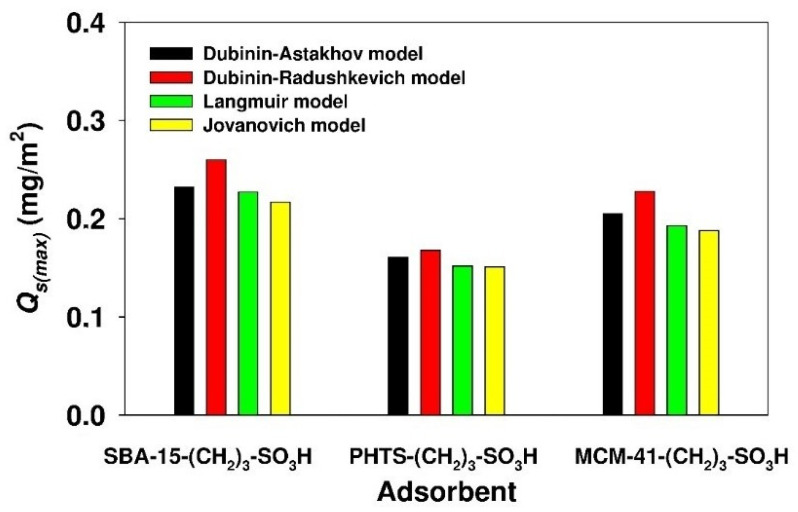
Surface area-normalized adsorption capacity of propyl-sulfonic acid-modified mesoporous adsorbents.

The values of *Q*_*s*(*max*)_ for given silicas are shown in Figure 7.

The value of the parameter was in the range of 0.15 to 0.16, 0.19 to 0.22 and 0.21 to 0.26 for PHTS, MCM-41 and SBA-15 modified silicas, respectively. As can be seen, the value of the parameter was comparable for SBA-15-(CH_2_)_3_-SO_3_H and MCM-41-(CH_2_)_3_-SO_3_H samples. For PHTS-(CH_2_)_3_-SO_3_H silica, the value was insignificantly lower; this may be attributed to the aforementioned steric hindrance resulting from the presence of silica nanoparticles inside the mesopores. A similar phenomenon was observed for the adsorption of chlorhexidine onto PHTS and SBA-16 silicas [54].

The values of the mean adsorption energy of ticagrelor onto all silicas modified with sulfonic acid derivative calculated based on the Dubinin-Radushkevich and Dubinin-Astakhov models significantly exceeded the value of 8 kJ/mol. This indicates the chemical nature of the interactions between the drug molecules and the surface of the modified silica [57,58]. However, the highest values of adsorption energy (12.8 kJ/mol, Dubinin-Astakhov model) were noted for the adsorption of ticagrelor onto modified SBA-15 and PHTS samples. Consistently, the highest values of Langmuir constant *K_L_* parameter were observed for these two types of silica.

The value of *β*, calculated from the Redlich-Peterson model, was in the range of 0.904 to 0.955. The obtained value of the parameter close to unity becomes the Redlich-Peterson equation, similar to the Langmuir-type isotherm [59].

Based on the *Q*_*ads*(*max*)_ value, the relationship between the amount of adsorbed ticagrelor to the number of propyl-sulfonic acid groups was also determined. For this purpose, the following equation was employed [55]:

(18) $$\frac{n_{TGR}}{n_{-{\left(CH_{2}\right)}_{3}-SO_{3}H}}=\frac{Q_{ads(\max)}\times 10^{-3}}{Q_{-{\left(CH_{2}\right)}_{3}-SO_{3}H}\times M_{TGR}}$$

where *n_TGR_* and *n_-(CH_2_)_3_-SO_3_H_* describe the number of moles of ticagrelor and propyl-sulfonic acid groups, respectively; *Q_-(CH_2_)_3_-SO_3_H_* represent the content (mol/g) of propyl-sulfonic acid groups in the adsorbent; *Q*_*ads*(*max*)_ represents the maximum adsorption capacity of silica (mg/g) calculated from given isotherm and *M_TGR_* is the molar weight (g/mol) of ticagrelor molecule.

**Figure 8:**
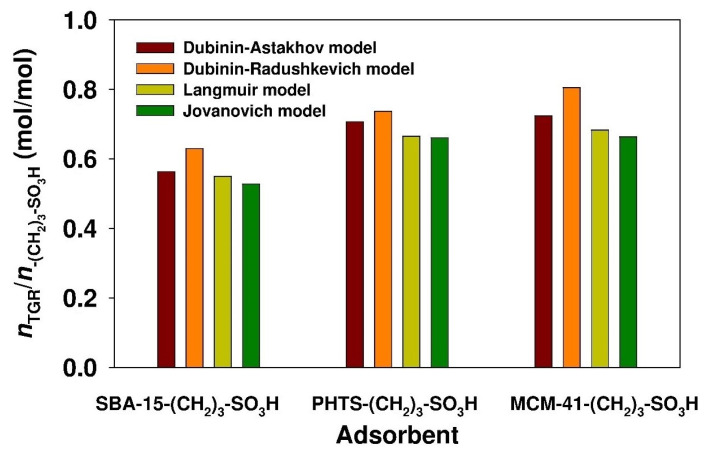
Molar ratio of ticagrelor molecules to propyl-sulfonic acid group content based on *Q*_*ads*(*max*)_ value parameter calculated from non-linear fitting analysis.

The results of aforementioned calculations are presented in Figure 8. 

The values of the parameter were in the range of 0.53 to 0.63, 0.66 to 0.73 and 0.66 to 0.81 for the SBA-15-(CH_2_)_3_-SO_3_H, PHTS-(CH_2_)_3_-SO_3_H and MCM-41-(CH_2_)_3_-SO_3_H adsorbents, respectively. The modified SBA-15 adsorbent revealed a slightly lower *n*_TGR_/*n*_-(CH_2_)_3_-SO_3_H_ value compared to other silicas. The values of the parameter show that ca. 60% of the acidic adsorption sites are accessible for ticagrelor (assuming that one sulfonic group binds with one molecule of ticagrelor). The binding of ticagrelor onto the modified mesoporous siliceous adsorbents may be due to interactions of sulfonic functions anchored at the silica surface with the basic moiety of ticagrelor molecule. Ticagrelor possesses an acido-basic character with pKa of 13.48 and 2.28 for acid and basic function, respectively [60]. Similar n_adsorbate_/n_-(CH_2_)_3_-SO_3_H_ values were observed in the case of the adsorption of boldine onto propyl-sulfonic acid-derivative modified SBA-15 silica [59]. However, for modified-PHTS silica, the degree of exploitation of acidic adsorption sites was higher for ticagrelor than for boldine (ca. 40%) [61]. 

**Figure 9:**
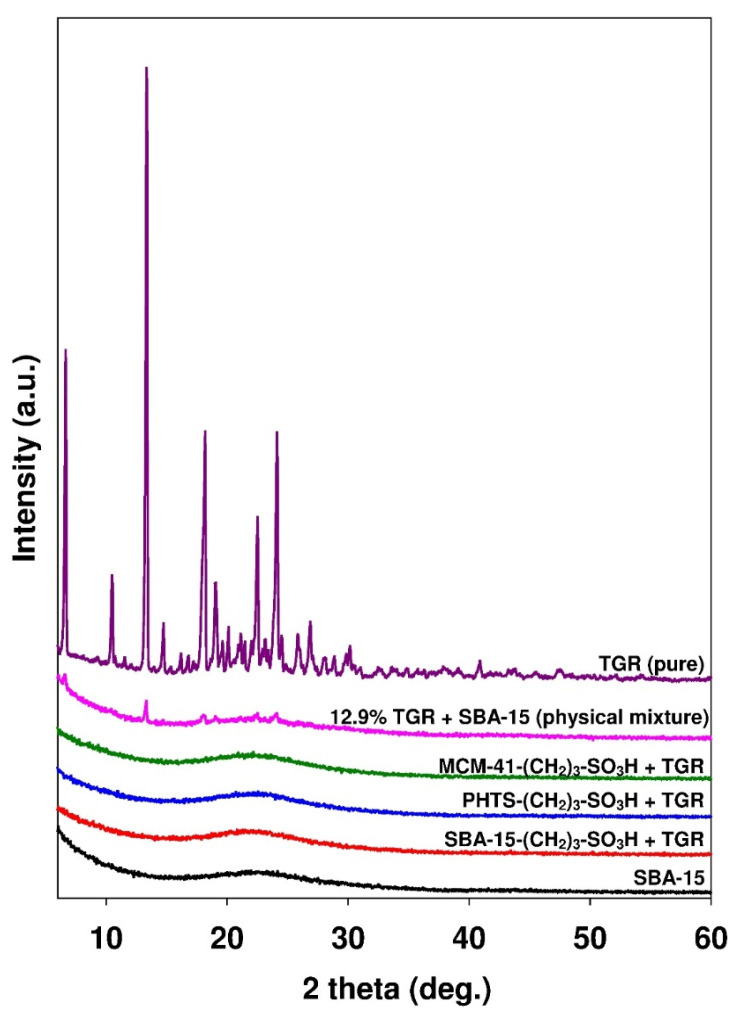
XRD diffraction patterns (wide-angle) of ticagrelor, physical mixture of ticagrelor with mesoporous silicas and mesoporous silica loaded with ticagrelor.

The wide angle XRD patterns of propyl-sulfonic acid derivative-modified silicas with adsorbed ticagrelor are presented in Figure 9. 

**Figure 10:**
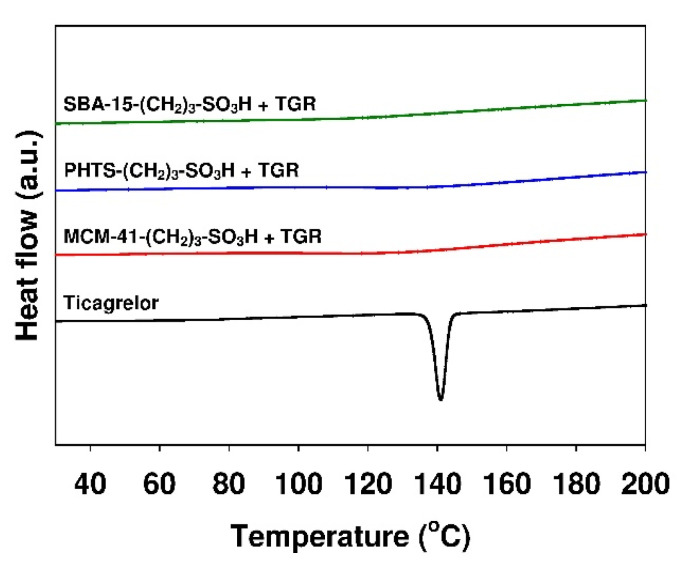
DSC curves of various mesoporous silicas loaded with ticagrelor.

The diffraction patterns of the physical mixture of pure crystalline ticagrelor with SBA-15 mesoporous silica (containing 12.9% of active substance), pure crystalline ticagrelor and nonmodified mesoporous adsorbent are shown for comparison. The physical mixture exhibited reflexes corresponding to the crystalline phase of pure ticagrelor localized at 2 theta diffraction angle at ~6.5, ~13.3, ~18.1, ~22.0 and ~24.0°. The diffractogram of pure amorphous silica revealed no reflexes. The lack of diffraction peaks in ticagrelor-containing samples indicated that the amorphous state of the active substance adsorbed onto the mesoporous matrices. Similar observations were described by Charnay et al. [62] in their work on the adsorption of ibuprofen onto MCM-41 material. The data would seem to suggest that ticagrelor adsorption occurred due to molecular dispersion [63,64,65]. The amorphous nature of the adsorbed ticagrelor was also confirmed using differential scanning calorimetry (DSC) analysis. In the DSC curve of pure ticagrelor presented in Figure 10, an endothermic peak at ca. 141.0 °C (minimum) can be observed. In the case of all ticagrelor-loaded mesoporous samples, the peak related to the melting of active substance cannot be distinguished.

### 3.4. Release Studies

**Figure 11:**
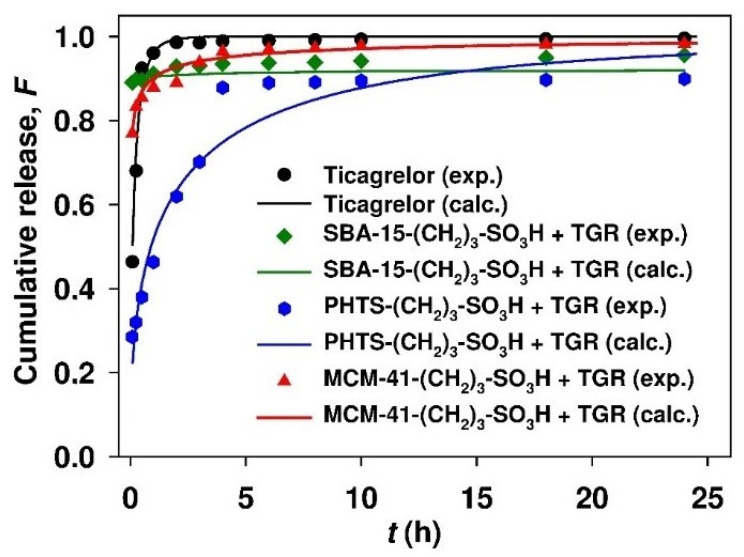
Profiles of cumulative release of ticagrelor from various mesoporous carriers. Medium: acetate buffer (pH 4.5) containing 0.5% (m/v) SDS addition.

The cumulative release profiles of ticagrelor from sulfonic acid derivative-functionalized mesoporous SBA-15, PHTS and MCM-41 silicas in acetate buffer (pH 4.5) containing 0.5% (m/v) SDS are presented in Figure 11. The dissolution profile of crystalline ticagrelor is shown for comparison. The amount of ticagrelor loaded in the prepared dosage forms varied from 6.5 to 15.4% (see Table 4).

As illustrated in Figure 11, at pH 4.5, modified SBA-15 and MCM-41 carriers accelerated the drug dissolution process, compared to the dissolution rate of free crystalline ticagrelor. Intriguingly, modified PHTS silica slowed the drug release kinetics compared to other siliceous adsorbents and to the dissolution rate of crystalline ticagrelor. The prolonged release of ticagrelor from the latter may be due to the significant microporosity of PHTS and from hindered diffusion of the drug from the microporous spaces [36]. The results shown in Figure 11 also indicate that all siliceous carriers exhibited a burst effect in the initial phase of release.

### 3.5. Ticagrelor Release Modeling

The parameters of Weibull Equations (13) determined for three mesoporous carriers and crystalline ticagrelor are summarized in Table 4.

Unambiguously, the results of release studies performed in acetate buffer (pH 4.5) demonstrated the accelerated dissolution of ticagrelor from modified SBA-15 and MCM-41 mesoporous adsorbents, as compared to dissolution rate of pure drug. Low *b* (*b* < 1) values indicate that the dissolution curve is parabolic with a higher initial slope, and after that, consistent with the exponential [48]. The highest scale parameter values in the Weibull equation were observed for the release of ticagrelor from PHTS-(CH_2_)-SO_3_H silica. This correlates with the most prolonged release of active substance from this carrier.

The time necessary to dissolve 50% and 90% of ticagrelor from mesoporous adsorbents at pH 4.5 increased in the following order: SBA-15-(CH_2_)_3_-SO_3_H < MCM-41-(CH_2_)_3_-SO_3_H < PHTS-(CH_2_)_3_-SO_3_H. The latter revealed the slowest release of the active agent. It should be noted that PHTS-(CH_2_)-SO_3_H adsorbent was also characterized by the highest adsorption energy value of ticagrelor (13.0 kJ/mol) among all the examined silicas (Dubinin-Radushkevich adsorption model), which may partially explain the stronger bonding of the drug with this carrier. PHTS silica also revealed the highest Langmuir and Temkin isotherm parameter values, which effectively describe the chemisorption processes. However, the calculated values were only slightly higher compared to SBA-15 silica. The prolonged release of ticagrelor from the propyl-sulfonic acid-modified PHTS carrier can be mainly ascribed to the nature of the material itself. On the one hand, the silica particles present in the mesoporous channels of this material hinder the modification and adsorption process. On the other hand, they may also contribute to the retardation of the release process from PHTS silica, compared to the other examined carriers.

It can be clearly seen that under the applied experimental conditions, the sulfonic acid derivative-modified SBA-15 and MCM-41 mesoporous carriers accelerated, whereas modified PHTS silica slowed down, the process of ticagrelor dissolution, compared to the dissolution rate of the crystalline drug.

## 4. Conclusions

SBA-15, PHTS and MCM-41 materials modified with propyl-sulfonic acid groups exhibited good adsorption properties towards ticagrelor. The process of ticagrelor adsorption onto functionalized carriers was more efficient in acetonitrile than in 2-propanol. The amount of adsorbed ticagrelor (plateau) on the examined silicas was in the range of 83 to 220 mg/g, and increased in the following order: PHTS-(CH_2_)_3_-SO_3_H < SBA-15-(CH_2_)_3_-SO_3_H, MCM-41-(CH_2_)_3_-SO_3_H. The adsorption mechanism of ticagrelor is the result of chemical interactions between the mesoporous carrier and the drug molecule. The obtained results indicate that ca. 60% of active sites on the siliceous surface were occupied by ticagrelor. At pH 4.5, the dissolution rate of ticagrelor from functionalized SBA-15 and MCM-41 carriers was markedly accelerated, compared to the dissolution kinetics of the crystalline drug. Meanwhile, modified PHTS sorbent led to the prolonged release of ticagrelor. It can be concluded that SBA-15 and MCM-41 mesoporous molecular sieves functionalized with propyl-sulfonic acid functions are promising matrices for the adsorption and release of ticagrelor.

## Figures and Tables

**Table 1:** Textural properties of nonmodified mesoporous materials.

Parameter	Adsorbent		
	SBA-15	PHTS	MCM-41
BET surface area (m^2^/g)	770	650	1160
BJH pore volume (cm^3^/g) ^a^	0.97	0.70	0.65
Pore diameter (nm)^a^	5.9	5.5	4.0
Micropore volume (cm^3^/g) ^b^	0.101	0.115	-
Micropore area (m^2^/g)^b^	204	220	-

^a^ Calculated from desorption branch of N_2_ isotherm. ^b^ Calculated from t-plot method (de Boer method).

**Table 2:** Textural properties of propyl-sulfonic acid-modified mesoporous materials.

Parameter	Adsorbent		
	SBA-15-(CH_2_)_3_-SO_3_H	PHTS-(CH_2_)_3_-SO_3_H	MCM-41-(CH_2_)_3_-SO_3_H
Amount of functional groups, *Q*_-(CH_2_)_3_-SO_3_H_ (mol/g) ^a^	4.75 × 10^−4^	2.03 × 10^−4^	5.72 × 10^−4^
BET surface area (m^2^/g)	603	465	1056
BJH pore volume (cm^3^/g)^b^	0.85	0.55	0.37
Pore diameter (nm) ^b^	5.8	5.1	3.9
Micropore volume (cm^3^/g) ^c^	0.053	0.073	-
Micropore area (m^2^/g) ^c^	105	139	-

^a^ Calculated from elemental analysis (sulfur content) ^b^ Calculated from desorption branch of N_2_ isotherm ^c^ Calculated from t-plot method (de Boer method).

**Table 3:** Isotherm parameters calculated from a nonlinear fitting analysis of ticagrelor adsorption onto propyl-sulfonic acid modified mesoporous silica.

Adsorption Model	Parameter	Adsorbent		
		SBA-15-(CH_2_)_3_-SO_3_H	PHTS-(CH_2_)_3_-SO_3_H	MCM-41-(CH_2_)_3_-SO_3_H
Freundlich	*K_F_* (mg^1-1/n^dm^3/n^/g)	34.85	26.00	28.34
	*n_F_*	5.390	7.408	3.800
	ARE (%)	14.19	6.43	20.14
Jovanovich	*Q*_*J*(*max*)_ (mg/g)	131.1	70.1	198.4
	*K_J_* (dm^3^/mg)	6.752 × 10^−2^	6.671 × 10^−2^	2.560 × 10^−2^
	ARE (%)	13.21	10.49	10.37
Langmuir	*Q*_*L*(*max*)_ (mg/g)	136.6	70.6	204.1
	*K_L_* (dm^3^/mg)	8.122 × 10^−2^	8.545 × 10^−2^	3.428 × 10^−2^
	ARE (%)	8.05	7.81	5.19
Temkin	*K_T_* (dm^3^/mg)	2.829	2.926	0.637
	*b_T_* (J g/mol mg)	147.4	288.7	81.38
	ARE (%)	6.27	4.44	6.07
Dubinin-Radushkevich	*Q*_*DR*(*max*)_ (mg/g)	156.5	78.2	240.7
	*K_DR_* (mol^2^/J^2^)	3.320 × 10^−9^	2.980 × 10^−9^	4.763 × 10^−9^
	*E_DR_* (kJ/mol)	12.3	13.0	10.2
	ARE (%)	6.30	6.03	9.91
Dubinin-Astakhov	*Q*_*DA*(*max*)_ (mg/g)	140.1	75.0	216.3
	*K_DA_* (mol*^nDA^*/J*^nDA^*)	1.352 × 10^−14^	1.425 × 10^−10^	4.276 × 10^−15^
	*n_DA_*	3.258	2.313	3.414
	*E_DA_* (kJ/mol)	12.8	12.8	11.4
	ARE (%)	4.92	5.87	2.67
Redlich-Peterson	*K_RP_* (dm^3^/g)	14.59	9.299	8.269
	*a_RP_* (dm^3β^/mg^β^)	0.185	0.256	5.475 × 10^−2^
	*β*	0.923	0.904	0.955
	ARE (%)	1.84	3.46	1.59

**Table 4:** Weibull equation parameters calculated for ticagrelor release from various mesoporous matrices in acetate buffer pH = 4.5 with 0.5% SDS addition.

Parameter	Formulation (Dosage Form)			
	SBA-15-(CH_2_)_3_-SO_3_H	PHTS-(CH_2_)_3_-SO_3_H	MCM-41-(CH_2_)_3_-SO_3_H	Pure TGR
*a*	0.401	1.371	0.432	0.309
*b*	0.061	0.460	0.185	0.663
*t*_50%_ (h)	7.72 × 10^−10^	0.896	1.48 × 10^−3^	0.098
*t*_90%_ (h)	0.268	12.16	0.969	0.598
ARE (%)	0.37	7.55	1.10	1.59
Ticagrelor amount (%)	11.73	6.51	15.43	-

## List of Abbreviations and Symbols

*a*	scale parameter of Weibull equation
*a_RP_*	constant of Redlich-Peterson isotherm (dm^3β^/mg^β^)
ARE	average relative error (%)
*b*	shape parameter of Weibull equation
*b_T_*	Temkin constant related to the adsorption heat (J g/mol mg)
BET	Brunauer-Emmett-Teller isotherm
BJH	Barrett-Joyner-Halenda isotherm
*β*	exponential constant of Redlich-Peterson isotherm
*C*_0_	initial adsorbate concentration (mg/dm^3^)
*C_e_*	equilibrium adsorbate concentration (mg/dm^3^)
*C_s_*	solubility (mg/dm^3^)
DSC	differential scanning calorimetry
*E_ads_*	efficiency of adsorption (%)
*E_DA_*	adsorption energy calculated from Dubinin-Astakhov model (J/mol)
*E_DR_*	adsorption energy calculated from Dubinin-Radushkevich model (J/mol)
*F*	fraction of drug release
*F_calc._*	fraction of drug release (calculated)
*F_exp._*	fraction of drug release (experimental data)
*K_DA_*	constant of Dubinin-Astakhov isotherm related to the adsorption energy (mol^nDA^/J^nDA^)
*K_DR_*	constant of Dubinin-Radushkevich isotherm related to the adsorption energy (mol^2^/J^2^)
*K_F_*	Freundlich constant (mg^1-1/n^dm^3/n^/g)
*K_J_*	Jovanovich constant (dm^3^/mg)
*K_L_*	Langmuir constant (dm^3^/mg)
*K_RP_*	constant of Redlich-Peterson isotherm (dm^3^/g)
*K_T_*	Temkin binding constant (dm^3^/mg)
*m*	mass of adsorbent (g)
*M_TGR_*	molar weight of ticagrelor molecule (g/mol)
MPTMS	(3-mercaptopropyl)trimethoxysilane
*n_TGR_*	number of ticagrelor molecules (mol)
*n_DA_*	heterogeneity factor of Dubinin-Astakhov isotherm
*n_F_*	exponential constant of Freundlich equation
*n_-(CH_2_)_3_-SO_3_H_*	number of propyl-sulfonic acid groups (mol)
p/p_0_	relative pressure
*Q*_*ads*(*max*)_	maximum adsorption capacity calculated from given isotherm model (mg/g)
*Q*_*DA*(*max*)_	maximum adsorption capacity calculated from Dubinin-Astakhov isotherm (mg/g)
*Q*_*DR*(*max*)_	maximum adsorption capacity calculated from Dubinin-Radushkevich equation (mg/g)
*Q_e_*	amount of adsorbate in equilibrium solid state (mg/g)
*Q_e,calc._*	amount of adsorbate in equilibrium solid state (calculated) (mg/g)
*Q_e,exp._*	amount of adsorbate in equilibrium solid state (experimental data) (mg/g)
*Q*_*J*(*max*)_	maximum adsorption capacity calculated from Jovanovich equation (mg/g)
*Q*_*L*(*max*)_	maximum adsorption capacity calculated from Langmuir equation (mg/g)
*Q*_*s*(*max*)_	surface area-normalized adsorption capacity (mg/m^2^)
*Q*_-(CH_2_)_3_-SO_3_H_	content of propyl-sulfonic acid groups (mol/g)
*R*	gas constant (8.314 J/mol K)
*S_BET_*	specific surface area calculated from BET model (m^2^/g)
STP	standard temperature and pressure
*T*	absolute temperature (Kelvin)
*t*	time (h)
TEM	transmission electron microscopy
TEOS	tetraethyl orthosilicate
TGR	ticagrelor

## References

[1] Dobesh P.P., Oestreich J.H. (2014). Ticagrelor: Pharmacokinetics, pharmacodynamics, clinical efficacy, and safety. Pharmacotherapy.

[2] Deeks E.D. (2011). Ticagrelor. A review of its use in the management of acute coronary syndromes. Drugs.

[3] Nylander S., Femia A.E., Scavone M., Bertsson P., Asztély A.-K., Nelander K., Löfgren L., Nilsson R.G., Cattaneo M. (2013). Ticagrelor inhibits human platelet aggregation via adenosine in addition to P2Y_12_ antagonism. J. Thromb. Haemost..

[4] Cattaneo M., Schulz R., Nylander S. (2014). Adenosine-mediated effects of ticagrelor. J. Am. Coll. Cardiol..

[5] Wittfeldt A., Emanuelsson H., Brandrup-Wognsen G., Van Giezen J.J.J., Jonasson J., Nylander S., Gan L.-M. (2013). Ticagrelor enhances adenosine-induced coronary vasodilatory responses in humans. J. Am. Coll. Cardiol..

[6] Nylander S., Schulz R. (2016). Effects of P2Y_12_ receptor antagonists beyond platelet inhibition—Comparison of ticagrelor with thienopyridines. Brit. J. Pharmacol..

[7] Storey R.F., Angiolillo D.J., Bonaca M.P., Thomas M.R., Judge H.M., Rollini F., Franchi F., Ahsan A.J., Bhatt D.L., Kuder J.F. (2016). Platelet inhibition with ticagrelor 60 mg versus 90 mg twice daily in the PEGASUS-TIMI 54 trial. J. Am. Coll. Cardiol..

[8] Gurbel P.A., Bliden K.P., Butler K., Antonino M.J., Wei C., Teng R., Rasmussen L., Storey R.F., Nielsen T., Eikelboom J.W. (2010). Response to ticagrelor in clopidogrel nonresponders and responders and effect of switching therapies. The RESPOND study. Circulation.

[9] Son G.-H., Na Y.-G., Huh H.W., Wang M., Kim M.-K., Han M.-G., Byeon J.-J., Lee H.-K., Cho C.-W. (2019). Systemic design and evaluation of ticagrelor-loaded nanostructured lipid carriers for enhancing bioavailability and anitplatelet activity. Pharmaceutics.

[10] Goto S., Huang C.-H., Park S.-J., Emanuelsson H., Kimura T. (2015). Ticagrelor vs. clopidogrel in Japanese, Korean and Taiwanese patients with acute coronary syndrome. Circ. J..

[11] Storey R.F., Bliden K.P., Patil S.B., Karunakaran A., Ecob R., Butler K., Teng R., Wei C., Tantry U.S., Gurbel P.A. (2010). Incidence of dyspnea and assessment of cardiac and pulmonary function in patients with stable coronary artery disease receiving ticagrelor, clopidogrel, or placebo in the ONSET/OFFSET study. J. Am. Coll. Cardiol..

[12] Parodi G., Storey R.F. (2014). Dyspnoea management in acute coronary syndrome patients treated with ticagrelor. Eur. Heart J. Acute Cardiovasc. Care.

[13] Parodi G., Xanthopoulou I., Bellandi B., Gkizas V., Valenti R., Karanikas S., Migliorini A., Angelidis C., Abbate R., Patsilinakos S. (2015). Ticagrelor crushed tablets administration in STEMI patients. J. Am. Coll. Cardiol..

[14] Vallet-Regí M., Rámilla A., del Real R.P., Pérez-Pariente J. (2001). A new property of MCM-41: Drug delivery system. Chem. Mater..

[15] Meynen V., Cool P., Vansant E.F. (2009). Verified syntheses of mesoporous materials. Microporous Mesoporous Mater..

[16] Díaz A., López T., Manjarrez J., Basaldella E., Martínez-Blanes J.M., Odriozola J.A. (2006). Growth of hydroxyapatite in a biocompatible mesoporous ordered silica. Acta Biomater..

[17] Liu X., Zhu I., Zhao T., Lan J., Yan W., Zhang H. (2011). Synthesis and characterization of sulfonic acid-functionalized SBA-15 for adsorption of biomolecules. Microporous Mesoporous Mater..

[18] Moritz M., Łaniecki M. (2012). SBA-15 mesoporous material modified with APTES as the carrier for 2-(3-benzoylphenyl)propionic acid. Appl. Surf. Sci..

[19] Hu Y., Wang J., Zhi Z., Jiang T., Wang S. (2011). Facile synthesis of 3D cubic mesoporous silica microspheres with a controllable pore size and their application for improved delivery of a water-insoluble drug. J. Colloid Interface Sci..

[20] Marques I.J., Vaz P.D., Fernandes A.C., Nunes C.D. (2014). Advantageous delivery of nifedipine from inorganic materials showing increased solubility and biobompatibility. Microporous Mesoporous Mater..

[21] Zhao Q., Wang T., Wang J., Zheng L., Jiang T., Cheng G., Wang S. (2011). Template-directed hydrothermal synthesis of hydroxyapatite as a drug delivery system for the poorly water-soluble drug carvedilol. Appl. Surf. Sci..

[22] Basaldella E.I., Legnoverde M.S. (2010). Functionalized silica matrices for controlled delivery of cephalexin. J. Sol-Gel Sci. Technol..

[23] Doadrio A.L., Doadrio J.C., Sànchez-Montero J.M., Salinas A.J., Vallet-Regí M. (2010). A rational explanation of the vankomycin release from SBA-15 and its derivative by molecular modelling. Microporous Mesoporous Mater..

[24] Moritz M. (2013). Solvent opitimization for niacinamide adsorption on organo-functionalized SBA-15 mesoporous silica. Appl. Surf. Sci..

[25] Wu Z., Jiang Y., Kim T., Lee K. (2007). Effects of surface coating on the controlled release of vitamin B_1_ from mesoporous silica tablets. J. Control. Release.

[26] Xu Z., Ji Y., Guan M., Huang H., Zhao C., Zhang H. (2010). Preparation and characterization of L-leucine-modified amphiprotic bifunctional mesoporous SBA-15 molecular sieve as a drug carrier for ribavirin. Appl. Surf. Sci..

[27] Tang Q., Xu Y., Wu D., Sun Y. (2006). pH-Controlled drug release from mesoporous silica tablets coated with hydroxypropyl methylcellulose phthalate. Mater. Res. Bull..

[28] Colila M., Izquierdo-Barba I., Vallet-Regí M. (2010). Phosphorus-containing SBA-15 materials as bisphosphonate carriers for osteoporosis treatment. Microporous Mesoporous Mater..

[29] Moritz M., Geszke-Moritz M. (2019). The effect of SBA-15 surface modification on the process of 18β-glycyrrhetinic acid adsorption: Modeling of experimental adsorption isotherm data. Materials.

[30] Baeza A., Guisasola E., Ruiz-Hernández E., Vallet-Regí M. (2012). Magnetically triggered multidrug release by hybrid mesoporous silica nanoparticles. Chem. Mater..

[31] Coll C., Mondragón I., Martínez-Máñez R., Sancenón F., Marcos M.D., Soto J., Amarós P., Pérez-Payá E. (2011). Enzyme-mediated controlled release systems by anchoring peptide sequences on mesoporous silica supports. Angew. Chem. Int. Ed..

[32] Yang S., Li N., Chen D., Qi X., Xu Y., Li H., Lu J. (2013). Visible-light degradable polymer coated hollow mesoporous silica nanoparticles for controlled drug release and cell imaging. J. Mater. Chem. B.

[33] Van der Voort P., Ravikovitch P.I., de Jong K.P., Neimark A.V., Janssen A.H., Benjelloun M., van Bavel E., Cool P., Weckhuysen B.M., Vansant E.F. (2002). Plugged hexagonal templated silica: A unique micro- and mesoporous composite material with internal silica nanocapsules. Chem. Commun..

[34] Li Q., Wu Z., Feng D., Tu B., Zhao D. (2010). Hydrothermal stability of mesostructured cellular silica foams. J. Phys. Chem. C.

[35] Bavel E.V., Cool P., Aerts K., Vansant E.V. (2004). Plugged hexagonal templated silica (PHTS): An in-depth study of the structural characteristics. J. Phys. Chem. B.

[36] Moritz M., Geszke-Moritz M. (2015). Mesoporous materials as multifunctional tools in biosciences: Principles and applications. Mater. Sci. Eng. C.

[37] Le T.-T., Elyafi A.K.E., Mohammed A.R., Al-Khattawi A. (2019). Delivery of poorly soluble drugs via mesoporous silica: Impact of drug overloading on release and thermal profiles. Pharmaceutics.

[38] Zhao D., Huo Q., Feng J., Chmelka B.F., Stucky G.D. (1998). Nonionic triblock and star diblock copolymer and oligomeric surfactant syntheses of highly ordered, hydrothermally stable, mesoporous silica structures. J. Am. Chem. Soc..

[39] Foo K.Y., Hameed B.H. (2010). Insights into the modeling of adsorption isotherm systems. Chem. Eng. J..

[40] Markovski J.S., Marković D.D., Ɖokić V.R., Mitrić M., Ristić M.Ɖ., Onjia A.E., Marinković A.D. (2014). Arsenate adsorption on waste eggshell modified by goethite, α-MnO_2_ and goethite/α-MnO_2_. Chem. Eng. J..

[41] Inglezakis V.J. (2007). Solubility-normalized Dubinin-Astakhov adsorption isotherm for ion-exchange systems. Microporous Mesoporous Mater..

[42] Hizal J., Demirçivi P., Karadirek Ş., Apak R. (2015). Investigation of individual and competitive adsorption of Cu(II), Cd(II), and Pb(II) on montmorillonite in terms of surface complexation and kinetic properties of Cu(II) adsorption. Desalin. Water Treat..

[43] Kundu S., Gupta A.K. (2006). Arsenic adsorption onto iron oxide-coated cement (IOCC): Regression analysis of equilibrium data with several isotherm models and their optimization. Chem. Eng. J..

[44] Garbacz G., Golke B., Wedemeyer R.-S., Axell M., Söderlind E., Abrahamsson B., Weitschies W. (2009). Comparison of dissolution profiles obtained from nifedipine extended release once a day products using different dissolution test apparatuses. Eur. J. Pharm. Sci..

[45] Phillips D.J., Pygall S.R., Cooper B.B., Mann J.C. (2012). Overcoming sink limitations in dissolution testing: A review of traditional methods and the potential utility of biphasic systems. J. Pharm. Pharmacol..

[46] European Pharmacopoeia 6.0. https://www.worldcat.org/title/european-pharmacopoeia/oclc/170932841.

[47] Tang J., Srinivasan S., Yuan W., Ming R., Liu Y., Dai Z., Noble C.O., Hayes M.E., Zheng N., Jiang W. (2019). Development of a flow-through USP 4 apparatus drug release assay for the evaluation of amphotericin B liposome. Eur. J. Pharm. Biopharm..

[48] Costa P., Lobo J.M.S. (2001). Modeling and comparison of dissolution profiles. Eur. J. Pharm. Sci..

[49] Zhang Y., Huo M., Zhou J., Zou A., Li W., Yao C., Xie S. (2010). DDSolver: An add-in program for modeling and comparison of drug dissolution profiles. AAPS J..

[50] Geszke-Moritz M., Moritz M. (2016). APTES-modified mesoporous silicas as the carriers for poorly water-soluble drug. Modeling of diflunisal adsortpion and release. Appl. Surf. Sci..

[51] Sing K.S.W., Everett D.H., Haul R.A.W., Moscou L., Pierotti R.A., Rouquérol J., Siemieniewska T. (1985). Reporting physisorption data for gas/solid systems with special references to the determination of surface area and porosity. Pure Appl. Chem..

[52] Kruk M., Jaroniec M., Ko C.H., Ryoo R. (2000). Characterization of the porous structure of SBA-15. Chem. Mater..

[53] Selvam P., Bhatia S.K., Sonwane C.G. (2001). Recent advances in processing and characterization of periodic mesoporous MCM-41 silicate molecular sieves. Ind. Eng. Chem. Res..

[54] Moritz M., Geszke-Moritz M. (2015). Mesoporous silica materials with different structures as the carriers for antimicrobial agent. Modeling of chlorhexidine adsorption and release. Appl. Surf. Sci..

[55] Geszke-Moritz M., Moritz M. (2016). Modeling of boldine alkaloid adsorption onto pure and propyl-sulfonic acid-modified mesoporous silicas. A comparative study. Mater. Sci. Eng. C.

[56] Giles C.H., MacEwan T.H., Nakhwa S.N., Smith D. (1960). Studies in adsorption. Part XI. A system of classification of solution adsorption isotherms, and its use in diagnosis of adsorption mechanisms and in measurement of specific surface areas of solids. J. Chem. Soc..

[57] Iriel A., Bruneel S.P., Schenone N., Cirelli A.F. (2018). The removal of fluoride from aqueous solution by a lateritic soil adsorption: Kinetic and equilibrium studies. Ecotoxicol. Environ. Saf..

[58] Cestari A.R., Vieira E.F.S., Vieira G.S., Almeida L.E. (2007). Aggregation and adsorption of reactive dyes in the presence of an anionic surfactant on mesoporous aminopropyl silica. J. Colloid Interface Sci..

[59] Ho Y.-S., Ofomaja A.E. (2006). Biosorption thermodynamics of cadmium on coconut copra meal as biosorbent. Biochem. Eng. J..

[60] Remko M., Remková A., Broer R. (2016). A comparative study of molecular structure, pKa, lipophilicity, solubility, absorption and polar surface area of some antiplatelet drugs. Int. J. Mol. Sci..

[61] Geszke-Moritz M., Moritz M. (2017). Use of mesoporous silica modified with a sulfonic acid-derivative as adsorbent for boldine. Przem. Chem..

[62] Charnay C., Bégu S., Tourné-Péteilh C., Nicole L., Lerner D.A., Devoisselle J.M. (2004). Inclusion of ibuprofen in mesoporous templated silica: Drug loading and release property. Eur. J. Pharm. Biopharm..

[63] Zhu W., Wan L., Zhang C., Gao Y., Zheng X., Jiang T., Wang S. (2014). Exloitation of 3D face-centered cubic mesoporous silica as a carrier for poorly water soluble drug: Influence of pore size on release rate. Mater. Sci. Eng. C.

[64] Wu C., Sun X., Zhao Z., Zhao Y., Hao Y., Liu Y., Gao Y. (2014). Synthesis of novel core-shell structured dual mesoporous silica nanospheres and their application for enhancing the dissolution rate of poorly water-soluble drugs. Mater. Sci. Eng. C.

[65] Zhang Y., Jiang T., Zhang Q., Wang S. (2010). Inclusion of telmisartan in mesocellular foam nanoparticles: Drug loading and release property. Eur. J. Pharm. Biopharm..

